# The *Dispanins*: A Novel Gene Family of Ancient Origin That Contains 14 Human Members

**DOI:** 10.1371/journal.pone.0031961

**Published:** 2012-02-20

**Authors:** Markus Sällman Almén, Nathalie Bringeland, Robert Fredriksson, Helgi B. Schiöth

**Affiliations:** Department of Neuroscience, Functional Pharmacology, Uppsala University, BMC, Uppsala, Sweden; University of South Florida College of Medicine, United States of America

## Abstract

The Interferon induced transmembrane proteins (IFITM) are a family of transmembrane proteins that is known to inhibit cell invasion of viruses such as HIV-1 and influenza. We show that the IFITM genes are a subfamily in a larger family of transmembrane (TM) proteins that we call *Dispanins*, which refers to a common 2TM structure. We mined the *Dispanins* in 36 eukaryotic species, covering all major eukaryotic groups, and investigated their evolutionary history using Bayesian and maximum likelihood approaches to infer a phylogenetic tree. We identified ten human genes that together with the known IFITM genes form the *Dispanin* family. We show that the *Dispanins* first emerged in eukaryotes in a common ancestor of choanoflagellates and metazoa, and that the family later expanded in vertebrates where it forms four subfamilies (A–D). Interestingly, we also find that the family is found in several different phyla of bacteria and propose that it was horizontally transferred to eukaryotes from bacteria in the common ancestor of choanoflagellates and metazoa. The bacterial and eukaryotic sequences have a considerably conserved protein structure. In conclusion, we introduce a novel family, the *Dispanins*, together with a nomenclature based on the evolutionary origin.

## Introduction

Membrane proteins are essential for the ability of all cellular organisms to respond and interact with their environment. Therefore they have attained large research interest and are one of the major groups of drug targets [1]. We have previously estimated that 27% of the human genes codes for alpha-helical membrane proteins and provided a comprehensive classification based on their function and evolutionary origin [2]. However, the identification and annotation of many membrane bound protein families is still being revised. We have during recent years worked on the annotation of both G protein-coupled receptors [3], [4] and solute carriers [5] and most of the genes of these large superfamilies now have a clear identity and annotation. There is however still large work to be done to clarify the identity, annotation and the evolutionary history of several families of membrane bound proteins. Establishing a rigid nomenclature based on evolutionary information and structural features of the predicted proteins facilitates prediction of the functional role of these genes that often have only have been studied in large gene or transcription consortia.

In previous studies we have found that membrane proteins with few transmembrane (TM) helices are less studied than other. This is particularly true for 2TM proteins where more than 70% of the about 700 proteins remained unclassified. Interestingly, we found evidence for several uncharacterized homologues to a small group of genes known as the Interferon-induced transmembrane proteins (IFITM) family. The IFITMs constitute a group with four human members (IFITM1–3, 5) that are found in a consecutive order on chromosome 11, having two transmembrane (2TM) helices. The IFITM4 gene is not present in human, but is located in proximity to the other four genes in the mouse genome. The IFITM1–3 proteins were identified 25 years ago as being upregulated by interferons (IFN) [6]. Recently they received considerable attentions as IFITM1–3 were found to prevent infection of a growing list of viruses such as HIV-1, SARS influenza A H1N1, West Nile and Dengue fever viruses [7], [8], [9], [10]. Hence, proteins of the IFITM family mediate part of the antiviral response orchestrated by IFNs. However, the IFITM family is also involved in other processes such as oncogenesis, bone mineralization (IFITM5) and germ cell development (IFITM1 and 3) and IFITM5 has not been identified as interferon-inducible [11], [12], [13], [14]. Although the biological roles of the IFITM genes are emerging, no thorough evolutionary analysis has been performed on this group.

In this study, we sought to infer the evolutionary history of the human IFITM genes and identify potential homologues. We mined 36 eukaryotic species, covering all major eukaryotic groups, and found that the IFITMs form a subfamily in a larger novel family that has ten human members in addition to the four IFITM genes. We propose *Dispanins* as a novel name for this family, which refers to their common 2TM structure. Further, we find that the eukaryotic *Dispanins* first appeared before the radiation of metazoa and that they branch out into four subfamilies (A–D). More surprisingly, we also discover that the *Dispanin*s are found in a large range of bacteria and in brown alga.

## Results

### Sequence retrieval

In total, we collected 87 eukaryotic IFITM homologues from *H. Sapiens* (14 genes) *M. musculus* (17 genes), *G. gallus* (6 genes), *X. tropicalis* (13 genes), *D. rerio* (7 genes), *P. marinus* (1 gene), *C. intestinalis* (1 gene), *B.floridae* (12 genes), *S. manosoni* (1 gene), *S. purpuratus* (9 genes), *N. vectensis* (4 genes) and *M. brevicollis* (1 gene). No IFITM genes could be detected in any of the remaining 24 analyzed proteomes, which covers all other major eukaryotic groups. The search of the nr database with HMMER did not get any hits outside metazoa except bacteria, choanoflagellates and the brown alga *Ectocarpus siliculosus* (2 genes). Nine genes were deemed as pseudogenes based on annotation and sequence analysis and removed from further analysis. In addition to the four previously identified human IFITM genes, ten novel human homologous genes were detected. These ten genes together with the four IFITM genes form a human gene family that we choose to call *Dispanins* based of their common 2TM structure. In UniProt we identified 65 annotated IFITM homologues from full bacteria proteome sets spread over seven different phyla (See table S1): *Acidobacteria* (2 genes), *Actinobacteria* (43 genes), *Cyanobacteria* (3 genes), TG1 (1 gene), *Bacteroidetes* (2 genes), *Firmicutes* (1 gene) and *Proteobacteria* (13 genes). Out of these, 46 bacterial sequences from 32 species were included for further analysis. No viral or Archaean genes were annotated as IFITM homologues in UniProt.

### Phylogenetic analysis and classification

**Figure 1:**
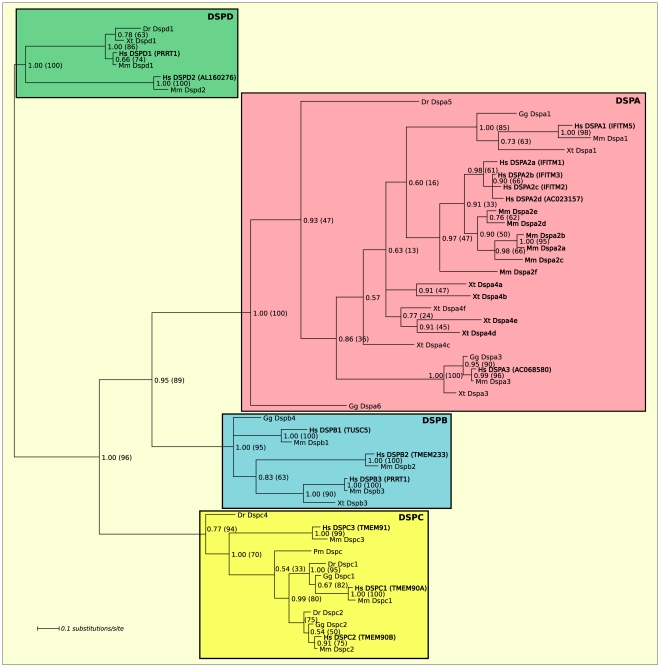
The phylogeny of the vertebrate *Dispanins*. The phylogenetic tree shows the propsed hierarchy of the eukaryotic *Dispanin* family. The four subfamilies (A–D) are marked with colors. Node support values are given as posterior probabilities (all nodes >0.5) and corresponding bootstrap values are given in parenthesis in percentage of 1000 bootstraps. Human sequences and sequences discussed in the manuscript are written in bold.

The phylogeny of the vertebrate *Dispanins* (Figure 1) allows the division of the *Dispanins* into four subfamilies A–D that are supported by strong confidence with respect to posterior probabilities (pp) or bootstraps (pp>0.75 and bs>90% for all nodes). We propose a common nomenclature for the *Dispanins* that are based on their subclass and a number (DSPA1 etc). The proposed names together with previous gene symbols and accession number can be found in Table S1.

The finding of two *Dispanin* homologs in the brown alga *E. siliculosus*, which is evolutionary distant to metazoa, and the single *Dispanin* in the close metazoan relative *M.brevicollis* are the two only non-metazoan eukaryotic *Dispanins*. A BLAST search gives that the *E. siliculosus* proteins have a higher similarity to metazoan family members (best hit E-value<10^−10^) than bacterial *Dispanins* and *M. brevicollis*. The *M. brevicollis Dispanin* share a conserved splice site with all metazoan family members, which suggest that the eukaryotic *Dispanins* first emerged in a common ancestor of *M. brevicollis* and the metazoan lineage. Within the metazoan lineage the *Dispanins* have been lost in at least two separate occasions, i.e. *T. adhaerens* and in the ecdysozoan lineage (*D. melanogaster* and *C. elegans*). The vertebrate *Dispanins* sort into subfamilies A–D (Figure 1). The DSPA subfamily has six human genes (DSPA1, DSPA2a–d and DSPA3) of which the DSPA2a–c corresponds to IFITM1–3 and DSPA1 to IFITM5. DSPA2d (AC023157) and DSPA3 (AC068580) are two novel identified genes, closely related to the IFITM family. The phylogenetic tree indicates that the DSPA2 genes have undergone an independent duplication in *H. Sapiens* and *M. musculus* and these were given a species specific nomenclature, e.g. DSPA2a–d in human. The *Dspa4*, *Dspa5*, *Dspa6* genes in the phylogenetic tree do not have any clear human orthologs. The DSPB subfamily is only found in tetrapoda and contains three human genes called DSPB1 (TUSC5), DSPB2 (TMEM233) and *DSPB3 (PRRT2)* whereas Dspb4 is only present in *G. gallus*. *DSPC1* (TMEM90A), DSPC2 (TMEM90B) and DSPC3 (TMEM91) make up the human DSPC subfamily, which is represented in all investigated vertebrates. The DSPD1 (PPRT1) gene is found in all the vertebrate species except the basal organism *P. marinus* whereas DSPD2 (AL160276) is mammalian specific.

**Figure 2:**
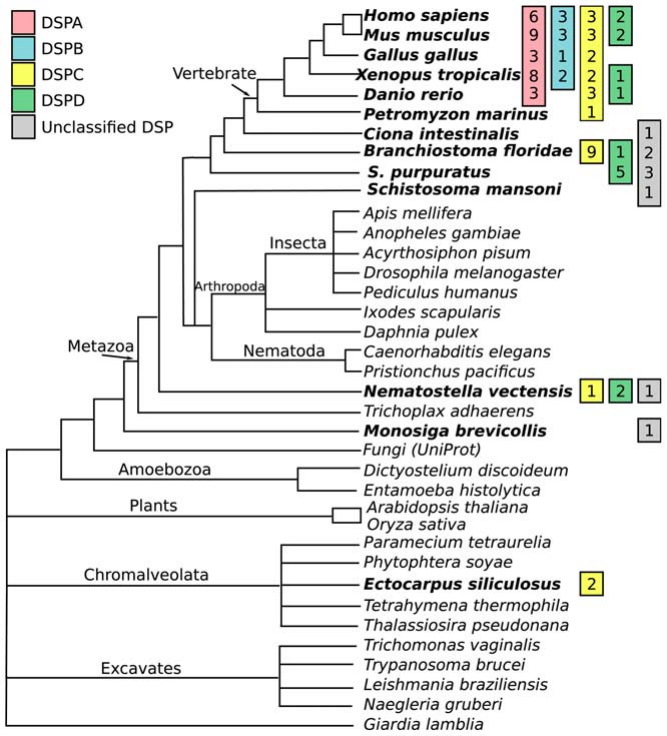
The evolutionary history of the *Dispanins*. The figure shows a schematic representation of the evolutionary history of the *Dispanin* family and its subfamilies (DSPA–D) and is based on the mining, phylogenetic analysis and evolutionary relationship of the species. The numbers at the branch ends represents the number of genes within each subfamily.

Five vertebrate genes and all invertebrate were excluded from the phylogenetic analysis and instead classified into subfamilies by using a BLAST approach (Table S1). Some genes could not unambiguously be classified into the vertebrate subfamilies: *C. intestinalis* (1 gene), *S. mansoni* (1 gene), *B. floridae* (2 genes), *S. purpuratus* (3 genes), *N. vectensis* (1 gene) and *M. brevicollis* (1 gene). By combining the results of the phylogenetic analysis and BLAST classification, we created a schematic overview of the organisms' gene repertoire and a schematic picture of the *Dispanin* family's evolutionary history, which suggests that the invertebrate *Dispanins* share more similarity towards the DSPC and D subfamilies than DSPA and B (Figure 2).

### Sequence analysis of the Dispanins

**Figure 3:**
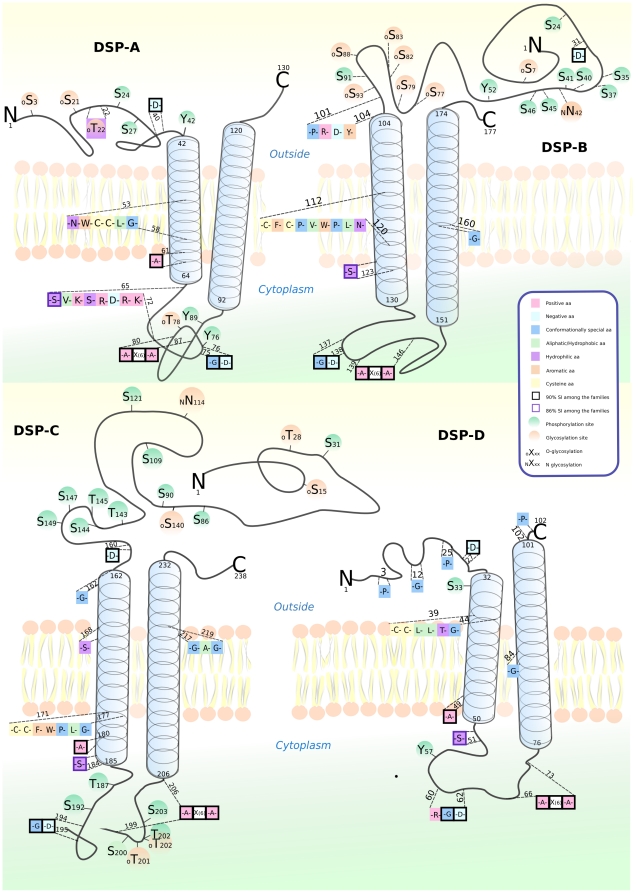
The protein features and topology of the *Dispanin* subfamilies. The picture shows the membrane topology and sequences features of a representative human member of each subfamily. Conserved motifs and residues are shown and those which have a sequence identity of more than 90% are framed in black and those with 80–90% sequence similarity are framed in blue. Predicted phosphorylation (Green) and glycosylation (orange) sites are shown.

All the members of the human *DSPA* are located on chromosome 11 except for DSPA2d which resides on chromosome 12. The genes of the other subfamilies are not enriched on any chromosome. Several features are common to all the eukaryotic *Dispanin* proteins (Figure 3). They comprise two transmembrane helices that are predicted between 20 and 30 amino acids in length with the second helices often being slightly longer. The *Dispanins* are rich in both glycosylation- and phosphorylation sites that predominantly are found on the N-terminus. The N-terminus is often long (>100 amino acids) compared to the C-terminal (<10 amino acids) and both are always oriented towards the outside of the cell.

**Figure 4:**
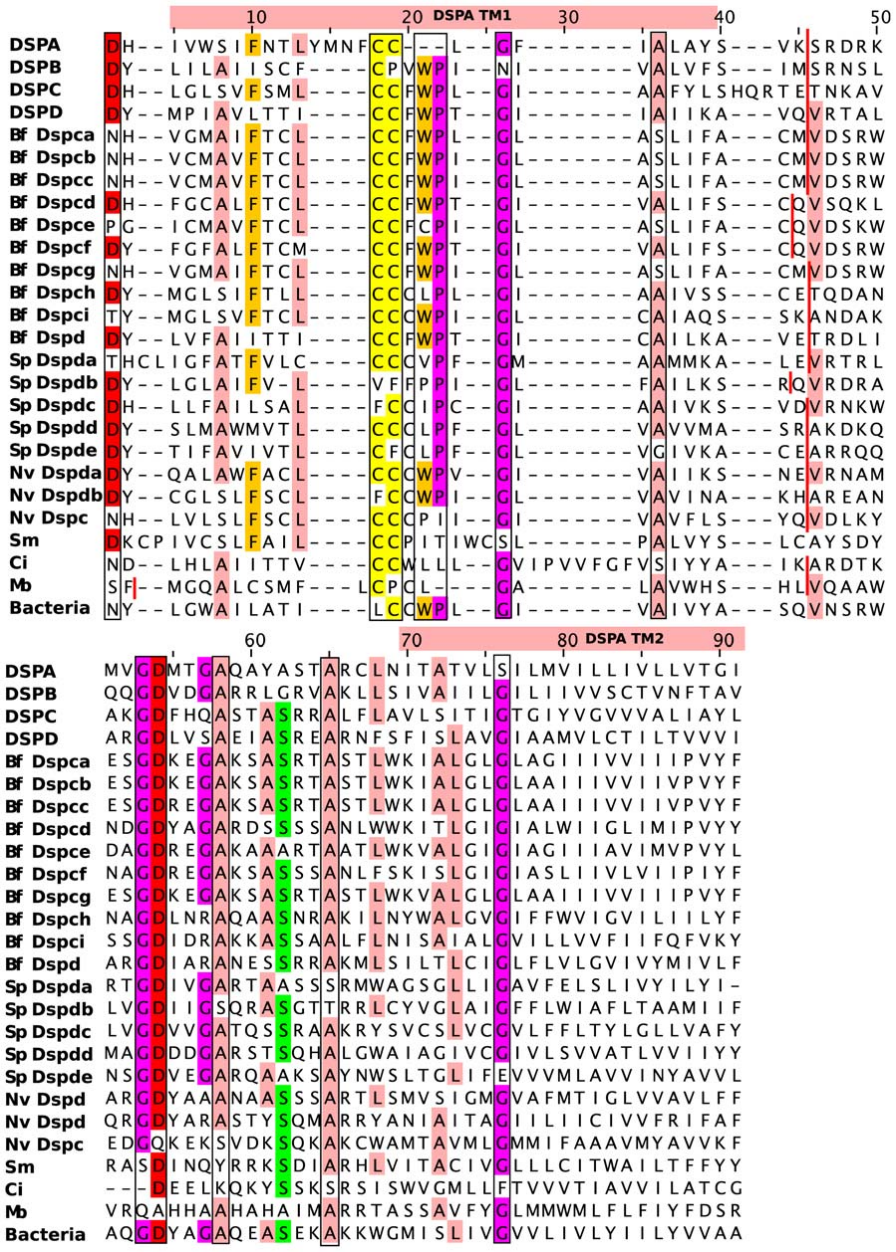
Multiple sequence alignment of the *Dispanins*. The multiple sequence alignment shows the conserved regions around the two transmembrane helices in the *Dispanins*. Consensus protein sequences of each vertebrate subfamily together with invertebrates, *M. brevicollis* (Mb) and a bacterial consensus sequences are included Conserved residues are coloured according to the Zappo scheme in Jalview and well conserved motifs are marked with a square. The conserved splice site between the two transmembrane helices is marked with a red line.

The *Dispanins* contain several conserved motifs (Figure 3 and 4), which are found both among eukaryotes and bacteria. The most conserved pattern is the G-D motif and the A-X(6)-A motif, both situated in the intracellular loop between the transmembrane helices that also is frequently rich in positive amino acids (K and R). The first helix is the most conserved with an alanine (A) residue and double cysteine C- C (C-F-C in the DSPB family) motif whereas a glycine (G) residue is the most conserved in the second helix. Another highly conserved motif, though only amongst the eukaryotes, is the single aspartic acid (D) on the N-terminus, flanking the first helix.

Analysis of the exon structure in the protein sequences was made for all eukaryotic *Dispanins* except *E. siliculosus* where no such information was found. The eukaryotic *Dispanins* have a conserved splice site in the intracellular loop that separates the two transmembrane helices into different exons (Figure 4). This site is only missing in the *S. mansoni Dispanin* and the mouse Dspa2f genes. The vertebrate proteins of the DSPA and DSPB subfamilies only have these two exons whereas the whole DSPC subfamily and the DSPD1 proteins have an additional exon that codes for their N-terminus. The DSPD2 proteins that only are found in mammals seem to have lost their N-termini exon. All the classified *B. floridae* and *S. purpuratus* sequences has the corresponding splice site in the N-terminus, whereas the *N. vectensis and M. brevicollis* proteins has 3–6 and 7 exons respectively.

## Discussion

We provide evidence that the four IFITM genes together with ten additional human genes, known as TUSC5, TMEM233, PRRT2, TMEM90A, DSPC2, TMEM90B, TMEM91, AC023157, AL160276 and AC068580, form a novel gene family that we call the *Dispanins*, which refers to the 2TM membrane topology that is common to all identified members. This family is the second largest 2TM family in the human genome, superseded only by the Inwardly rectifying potassium channel family that has 15 members [2]. Except for the 2TM memebrane topology the *Dispanins* are not homologous or share domains with any other 2TM proteins in the human genome and constitute a distinct gene family. We have discovered that this family is found in metazoan, the choanoflagellate *M. brevicollis* and the brown alga *E. siliculosus*, but not in other eukaryotes. Surprisingly it is widely present in bacteria where it is found in several different phyla such as *Actinobacteridae*, *Acidobacteria*, *Cyanobacteria*, *Bacteriodetes*, *Firmicutes* and *Proteobacteria*. The highest number of bacterial *Dispanins* is detected in *Actinobacteria* and *Proteobacteria*, which diverged around three billion years ago [15]. We find that *Dispanins* in eukaryotes and bacteria have high sequence similarities and share several conserved sequence motifs (Figure 4), which is strong evidence for a common evolutionary origin and possibly a functional relationship. As the family is found in several bacterial phyla we suggest that it first emerged in bacteria to later be introduced in eukaryotes through a horizontal gene transfer event. However, we were not able to construct a stable phylogenetic tree including bacterial and eukaryotic *Dispanins.* As the eukaryotic *Dispanins* only is widespread in metazoa it was unexpected to find the family in the evolutionary distant brown alga *E. siliculosus* (Figure 2). Our sequence analysis supports that all metazoan *Dispanins* have their origin in the common ancestor of *M. brevicollis* and metazoa as the choanoflagellate share a conserved splice site in the intracellular loop with nearly all metazoan *Dispanins* (Figure 4). However, the finding of the family in *E. siliculosus* suggests that the family has undergone two horizontal gene transfer events. As the *E. siliculosus Dispanins* are more similar to metazoan family members than *M. brevicollis* and bacteria we propose that the first horizontal gene transfer event was from bacteria to a common ancestor of choanoflagellates and metazoa followed by a second transfer between metazoa and brown alga.

During the course of metazoan evolution the *Dispanins* have expanded and diverged into four distinct subfamilies. However, it has also been lost in the basal metazoa *T. adhaerens* and the ecdysozoan lineage, which show that it is not essential for all metazoan life. Although we were unable to create a stable phylogenetic tree that include both vertebrate and invertebrate sequences BLAST searches suggest that the DSPC and D subfamilies are the oldest of the vertebrate subfamilies as the invertebrate sequences has higher resemblance to these two subfamilies (Figure 2). Moreover, the DSPC and D subfamilies forms a separate cluster from DSPA–B (Figure 1). Hence, the phylogenetic analysis suggests that the DSPA and B subfamilies have their origin close to the radiation of teleost, although DSPB have been lost in *D. rerio* (Figure 1 and 4). The DSPC family is found in two to three copies in all vertebrates and is the most widespread family as the BLAST classification suggest that it is present in two invertebrate species and *E. siliculosus*. In mouse, the family members are expressed predominantly in brain tissues (Dspc1/Tmem90a, Dspc2/Tmem90b) or ubiquitously (Dspc3/Tmem91). DSPC1 (TMEM90A) has been proposed to have a role in striatial functioning and the pathophysiology of Huntington's disease and is localized to the Golgi apparatus [16]. The DSPD family has been lost at several occasions, both in vertebrates and invertebrates (Figure 2). The mouse Dspd1 (Prrt1) gene is ubiquitously expressed with the highest expression in B-cells according to BioGPS [17]. However, no previous studies have been performed on the DSPD subfamily. The DSPB subfamily is found in *tetrapoda* and has three members in human and mouse. Mice expression profiles from BioGPS shows that Dspb3 (Prrt2) is exclusively expressed in brain tissues and that Dspb1 (Tusc5) is expressed in dorsal root ganglia and adipose tissues. In agreement with this expression data, Dspb1 (Tusc5) has been suggested to be involved in neural regulation of adipocyte differentiation and is regulated by PPARγ [18], [19]. The DSPA/IFITM subfamily is the most numerous and the mouse and human genes are all clustered in a consecutive manner on chromosome six and eleven, respectively. These regions share a conserved synteny (http://cinteny.cchmc.org/) and are flanked by the ATHL1 and B4GALNT4 genes on each side, which is strong evidence for the genes to have their origin in common evolutionary gene duplications. This is supported by the phylogenetic analysis (Figure 1) for DSPA1 (IFITM5) and DSPA3 (AC068580), which have orthologs in all *tetrapoda*. However, for the DSPA2 group (DSPA2A–D/IFITM1–3 and AC068580) the phylogeny suggests that *M. musculus* and *H. sapiens* have undergone independent expansions of the group. Rather than being created by independent gene duplications in the two species, it is possible that these genes are subject to concerted evolution, where paralogous genes within a species are more conserved towards each other than towards orthologs in other species. This phenomenon is most common in tandemly repeated genes, such as the DSPA2 group, and is believed to primarily be the result of recombination mechanisms [20]. Interestingly, also the Dspa4a-f genes of *X. tropicalis* seem to have undergone and independent expansion. However, the phylogeny is not strong enough to prove that the *Dspa4a-f* genes are orthologous to the mammalian DSPA2 genes (Figure 1). The DSPA/IFITM subfamily is the most well studied and is a multifunctional family of which its antiviral properties are best understood [8]. The family is expressed in many mouse tissues with the highest expression in mast cells, macrophages and osteoblasts according to BioGPS. We add two novel human members to this subfamily: DSPA2D, which is closely related to DSPA2C (IFITM3) and DSPA3, which forms a distinct cluster (Figure 1). Both these genes are poorly characterized. Microarray data from Array Express (www.ebi.ac.uk/arrayexpress/) shows that DSPA3 is upregulated by interferon after exposure of macrophages to interferon-gamma in a study (E-GEOD-5099) where DSPA2a–c (IFITM1–3) also were induced [21]. The mouse Dspa3 gene is like the other genes of this subfamily situated on chromosome seven, but is 1.5 Mbp away from the DSPA (IFITM) cluster where the other genes reside. Hence, *Dspa3* could explain the mild phenotypes and be responsible for the suggested functional redundancy that was found when deleting the whole DSPA (IFITM) loci [22].

The *Dispanin* family has several conserved motifs across subfamilies that are also detected in bacteria (Figure 4). One of the most prominent is the double cysteine motif (C-C) in the first transmembrane helix. This motif has recently been shown to undergo post-translational modification through S-palmitoylation in DSPA2C (IFITM3), which increases hydrophobicity [23]. Further, Yount and colleagues shows that the antiviral activity of DSPA2C (IFITM3) is dependent on this modification, which induces clustering of the proteins. As this motif is highly conserved, it is likely that S-palmitoylation is an important regulatory mechanism also among the other subfamilies. Intriguingly, this motif is also found in the bacterial *Dispanins* even though bacterial proteins do not undergo S-palmitoylation. Hence, the cysteine motif of the may have other means of structural and functional importance apart of from the S-palmitoylation.

In this study, we introduce the *Dispanin* family, of which the IFITM genes constitute a subfamily. In addition to the IFITM genes we identify 10 novel human *Dispanins* and investigate the family's evolutionary history and suggest that the eukaryotic members are descending from bacteria through a horizontal gene transfer. Thus, the expansion and diversification of *Dispanins* in vertebrates may reflect the evolution of a larger functional repertoire, which is a supported by the distinct expression profiles of the subfamilies. By identifying homologs to the IFITM genes and establishing the *Dispanins* as a family together with a solid detailed and evolutionary based nomenclature for the vertebrate genes, we provide a fundament for future functional characterization these genes.

## Methods

### Retrieval of protein sequences

The whole proteome dataset for the following eukaryotic species was included in the analysis: *Homo sapiens*, *Mus musculus*, *Gallus gallus*, *Xenopus tropicalis*, *Danio rerio*, *Petromyzon marinus*, *Drosophila melanogaster*, *Caenorhabditis elegans*, *Saccharomyces cerviciae*, *Schistosoma mansoni*, *Apis mellifera*, *Anopheles gambiae*, *Pediculus humanus*, *Ixodes scapularis*, *Daphnia pulex*, *Oryza sativa*, *Pristionchus pacificus*, *Acyrthosiphon pisum*, *Trypanosoma brucei*, *Leishmania braziliensis* and *Ciona instestinalis* were downloaded from Ensembl; *Strongylocentrous purpuratus* was downloaded from Spbase (www.spabase.org); *Branchiostoma floridae*, *Nematostella vectensis*, *Trichoplax adhaerens*, *Phytophtera soyae*, *Thalassiosira pseudonana*, *Naegleria gruberi* and *Monosiga brevicollis* were downloaded from the Joint Genome Institute; *Dictyostelium discoideum* was downloaded from dictyBase (www.dictybase.org); *Arabidopsis thaliana* was downloaded from TAIR (http://www.arabidopsis.org/); *Entamoeba histolytica* was downloaded from amoebaDB (http://amoebadb.org); *Paramecium tetraurelia* was downloaded from NCBI; *Tetrahymena thermophila* was downloaded from UniProt; *Trichomonas vaginalis* was downloaded from TrichDB (http://trichdb.org); *Giardia lamblia* was downloaded fromGiardiaDB (http://giardiadb.org).

All proteomes were searched against a local installation of the Pfam database (v.23) [24] using HMMER3 [25] and the script pfam_scan.pl, which was obtained from the Pfam ftp-site (ftp://ftp.sanger.ac.uk/pub/databases/Pfam/Tools/), with Pfam's default settings. In Pfam, the IFITM family is represented by a specific hidden Markov model [Pfam: P04505]. All the proteins that were assigned to this model in the Pfam-search were considered to be homologous to the IFITM family and were therefore included for further analysis. The script pfam_scan.pl uses the homology criterion set by the Pfam database, which is based on a manually curated gathering threshold for each model. The gathering threshold for PF04505 is a score of 20.6. The sequence datasets were controlled for annotated pseudogenes and transcript variants from the same gene. In the case of multiple transcript variants, the longest sequence was kept. The resulting non-redundant datasets were used for the analysis. The bacterial sequences were obtained by querying Uniprot (www.uniprot.org) for the Pfam ID [Pfam: PF04505]. Thereafter, the sequence set was downloaded by browsing by taxonomy and restricting it to species with a full proteome set. To assure that no lineages were missing in the selection of proteomes the nr protein dataset from NCBI was downloaded. The nr datasets contained 15,322,545 (20-09-11) protein sequences from a wide range of organisms. The dataset was searched against the PF04505 Pfam model using HMMER3 with default settings and sequences with a score above the Pfam gathering threshold (20.6) were deemed as homologous to IFITM.

### Multiple sequence alignment and phylogenetic analysis

Mafft-einsi was used, with default settings, to create a multiple sequence alignment (MSA) for the vertebrate protein sequences [26]. The MSAs were thereafter examined and refined in Jalview 2.5.1 [27], i.e. the sequences were trimmed and well conserved and aligned regions were kept, which included 82 aligned amino acid columns. Phylogenetic analysis was performed with a Bayesian approach implemented in MrBayes [28]. The following settings for the eukaryote proteins were adjusted: The analysis was run using a gamma shaped model for the variation of evolutionary rates across sites (rates = gamma) and the mixed option (aamodelpr = mixed) was used to estimate the best amino acid substitution model. We generated 5 000 000 trees and the Markov chain Monte Carlo analysis reached well below a standard deviation of split frequencies of 0.01. Each hundred tree was sampled from the mcmc run and the first 25% of the sampled trees were discarded (burnin = 0.25) to reassure a good sample from the posterior probability distribution. A consensus tree was built from the remaining 37 500 trees with the MrBayes *sumt* command using the 50% majority rule method. The *sump* command was used to assure that an adequate sample of the posterior probability distribution was reached during the mcmc procedure. To validate the phylogenetic inference with MrBayes a maximum likelihood method implmemented in RAxML was used [29]. The combined rapid bootstrapping and search for the best-scoring ML tree option (-f a) in RAxML was used to create 1000 bootstraps (-# 1000) using a gamma model of evolutionary rates and the JTT substitution model (-m PROTGAMMAJTT). The JTT substitution model was identified as the most suitable model in the Bayesian analysis and therefore selected for the maximum likelihood phylogeny. The consensus phylogenetic tree found with MrBayes was drawn in Dendroscope 3.0 and the bootstrap support values from RAxML were annotated on the corresponding nodes [30]. The phylogenetic tree was used to determine subfamilies by identifying clusters with a high posterior probability and bootstrap support. Invertebrate sequences were excluded from the phylogenetic analysis as they induced highly unstable topologies together with 1 *M. musculus*, *2 D. rerio* and 3 *X. tropicalis sequences*. These excluded sequences were categorized into their respective subfamilies by using a BLAST search towards the categorized sequences. The top five hits were examined to classify the invertebrate and excluded sequences into subfamilies. A sequence was assigned to the subfamily if four out of the five top hits are from the same subfamily.

### Sequence analysis

Several resources were used to identify the protein sequence features of the *Dispanins*. NetPhos 2.0 [31] identified potential phosphorylation sites. NetNGlyc 1.0 and NetOGlyc 3.1 [32] were used to find possible N- and O-glycosylation sites respectively. Transmembrane helix prediction was made using TMHMM 2.0 [33]. Motifs were found manually through Jalview 2.5.1 and the server MEME 4.4.0. Finally, EMBOSS:cons was used to create consensus sequences of the different Dispanin families that emerged from the phylogenetic analysis and the bacterial sequences. The consensus sequences were aligned together with the *M. brevicollis* and the invertebrate sequences. The resulting MSA was viewed and trimmed in Jalview and the conserved region around the TM helices was kept. Splice sites were detected in the eukaryotic *Dispanins* by studying their annotation in the respective databases and align them to their genome using BLAT at the UCSC Genome Browser website (http://genome.ucsc.edu).

## Supporting Information

Table S1 This is a record of all identified Dispanins together with their accession numbers, nomenclature and species belonging.
(XLS)
